# Special Immunization Service: A 14-year experience in Italy

**DOI:** 10.1371/journal.pone.0195881

**Published:** 2018-04-12

**Authors:** Daniele Donà, Susanna Masiero, Sara Brisotto, Lorena Gottardello, Rebecca Lundin, Eleonora Borgia, Federica Visentin, Liviana Da Dalt

**Affiliations:** 1 Division of Pediatric Infectious Diseases, Department for Woman and Child Health, University of Padua, Padua, Italy; 2 Pediatric Emergency Unit, Department for Woman and Child Health, University of Padua, Padua, Italy; 3 Department of Hygiene and Public Health, University of Padua, Padua, Italy; 4 PENTA Foundation, Padua, Italy; Universita degli Studi di Catania, ITALY

## Abstract

**Background:**

Concerns regarding vaccine safety are increasing along with lack of compliance to vaccination schedules. This study aimed to assess vaccination-related risks and the impact of a Special Immunization Service (SIS) at the Pediatric Emergency Department (PED) of Padua on vaccination compliance among participants.

**Materials and methods:**

This retrospective cohort study included all children attending the SIS from January 1^st^ 2002 to December 31^st^ 2015. The Service is divided into a clinic (SIS-C) where all referred children undergo a pre-vaccination visit and an area within the Pediatric Emergency Department (SIS-PED) where children are vaccinated if indicated. During each SIS-C visit, age, gender, admission criteria and scheduled vaccinations were recorded, with any vaccine-related adverse events captured during SIS-PED visits. Follow-up was conducted to evaluate vaccination plan completion.

**Results:**

359 children received 560 vaccine administrations (41.3% MMR/MMRV, 17.5% hexavalent) at the SIS during the 14 year study. Admission criteria were adverse events after previous vaccination (immediate, IgE/not IgE mediated, and late) in 27.2% of cases, non-anaphylactic allergies (mostly egg allergy) in 42.7% and anaphylaxis in 10.3%. After vaccination, 15/560 (2.7%) mild adverse events were observed. 96.3% of children vaccinated at least once at the SIS-PED and available for follow-up completed their vaccination plan, in contrast to 55.5% of children referred to the SIS-C who were not vaccinated in SIS-PED.

**Conclusions:**

For children referred to SIS-C and available for follow-up, vaccination in SIS-PED was associated with more frequent completion of vaccination plans, indicating a benefit of the service to vaccine coverage. The low number and mild severity of adverse events reported after vaccination of high-risk children in SIS-PED attest to the safety of the service

## Introduction

Vaccine administration is one of the greatest achievements of biomedical science and public health, and among the most effective medical procedures, preventing two to three million deaths every year [1]. It also provides indirect protection or ‘herd immunity’ to those who cannot be vaccinated due to age or specific diseases or treatments [2].

The safety of vaccines in the pediatric population is of the utmost importance for parents and healthcare workers, especially in countries in which vaccine-preventable diseases are rare [3]. As with any medical procedure, some risks are involved; minor reactions are common, while serious reactions are rare [4], with anaphylaxis occurring in one in a million vaccine doses [5].

Some children are at higher risk of vaccine-related adverse events (AEs) due to reactions to previous vaccinations, egg allergies, or anaphylaxis [6].

With vaccination increasingly questioned by concerned parents and compliance to vaccination schedules falling [7–10], a Special Immunization Service (SIS) was established in 2002 at the Pediatric Emergency Department (PED) in Padua to monitor and address immunization risks.

SIS in other settings have played a central role in improving adherence to regional vaccination plans and increasing immunization coverage [11–13].

The primary aim of this study is to describe SIS referrals and visits in Padua and AEs observed after vaccinations over a 14-year period.

The secondary aim is to assess vaccination plan compliance after SIS attendance.

## Materials and methods

This is a retrospective cohort study including all referrals and visits to the Padua SIS from January 1^st^ 2002 to December 31^st^ 2015.

### Setting

The SIS, established on January 1^st^ 2002 in the PED of Padua University Hospital, is divided into a SIS-Clinic (SIS-C) in which a pre-vaccination evaluation takes place and an area within the PED (SIS-PED) where vaccinations are administered when indicated.

**Fig 1:**
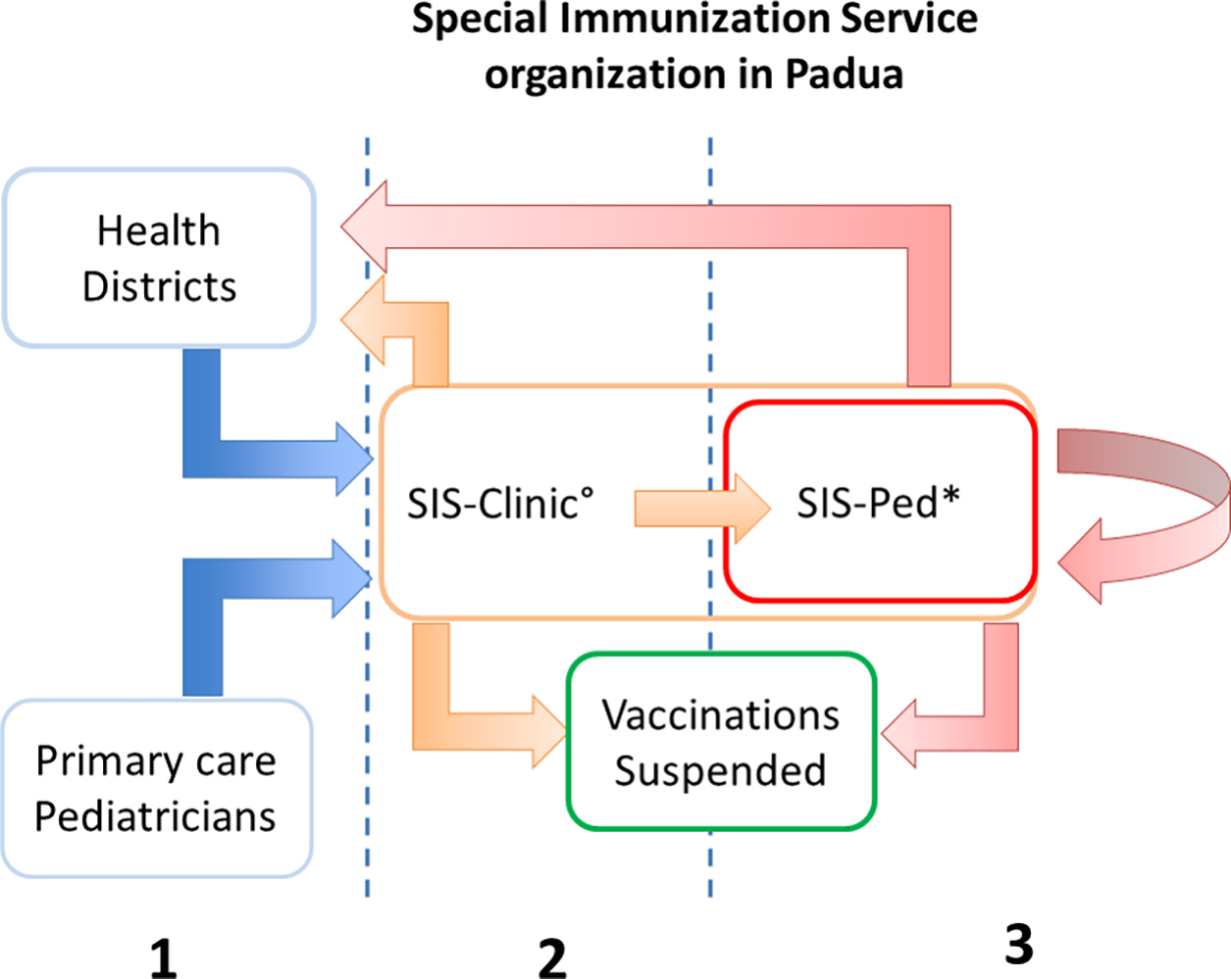
Special Immunization service organization in Padua. *population for primary aim of this study. °population for secondary aim of this study.

Children are referred to our Service by primary care pediatricians or local Health Districts of Padua if considered at high risk for vaccine-related AEs or for non-adherence to vaccination schedules due to parental reluctance (Fig 1 section 1).

At the SIS-C, each child is seen by a pediatrician with sub-specialty training in immunization techniques who determines, according to clinical history or diagnostic tests performed (RAST, Prick test, Prick by Prick), whether to suspend the vaccination plan or to proceed. Patients advised to continue their vaccination schedule may be vaccinated in the SIS-PED or their local Health Districts according to their risk of AEs. Decisions are made following the most recent Guidelines of the Italian Higher Institute of Health [6] and National Guidelines for the immunization in a SIS [14], and taking into consideration parents' concerns (Fig 1 section 2).

In the SIS-PED, each child is evaluated by a pediatrician to exclude acute illness and preparations are made to respond to possible AEs prior to vaccination. Afterwards, children remain in the SIS-PED for a three-hour observation period, which can be extended as deemed necessary by the attending pediatrician.

Following SIS-PED vaccination, each child is advised to complete their vaccination plan at the SIS-PED or local Health District, or to suspend their vaccination plan on the basis of any AE and accounting for parental preference (Fig 1 section 3).

### Data collection

Data were collected retrospectively and entered into an Excel database.

A unique survey code was assigned to each patient and all data were anonymized to guarantee data privacy.

This study was approved by the Institutional Review Board of the Department for Woman and Child Health at the University of Padua.

### Outcomes

#### Primary aim

For all children vaccinated in SIS-PED the following data were collected:

- Motivation for referral to SIS-C, including:
  - allergies: both anaphylactic and non-anaphylactic reactions (egg, dairy, drug, multiple allergies);
  - AEs after previous vaccinations: immediate or delayed reactions and IgE mediated or not;
  - other reasons: parents who chose not to vaccinate their children (until vaccination became mandatory in 2007), chronic conditions such as hemophilia, cutaneous mastocytosis, thrombocytopenia, epilepsy and rare genetic syndromes.
- Type of vaccine(s) administered
- AEs occurring during post-vaccination observation period or reported by parents via telephone after leaving SIS-PED.

#### Secondary aim

SIS-PED admissions and total vaccinations in the Padua urban area were compared using the ULSS 6 EUGANEA Prevention Department database, where all administered vaccinations in the metropolitan area are registered. This database was also used to follow up all children visiting SIS-C regardless of whether they were subsequently seen in SIS-PED.

Compliance was categorized as vaccination plan completed at the SIS, vaccination plan completed at the Health District or vaccination plan not completed.

Vaccination plan completion was defined as the accumulation of the required number of doses of all required immunizations by the specified age regardless of the timing of administration. Current Italian vaccination schedule at time of follow-up was used to determine required vaccines, doses and timelines (Table 1).

**Table 1:** Routine childhood and adolescent vaccines in Italy.

Vaccine	Birth	3rd month of age	5th month of age	7th month of age	9th month of age	13th month of age	14th month of age	15th month of age	5–6 years	11–12 years	14–15 years
**DTP**		DTaP	DTaP			DTaP			DtaP or dTap		dTap
**DT**											
**IPV**		IPV	IPV			IPV			IPV		IPV
**HepB**	HepB	HepB	HepB			HepB					
**HiB**		HiB	HiB			HiB					
**MMRV**							MMRV or MMR+Var		MMRV		
**Varicella**											Varicella
**PCV**		PCV	PCV					PCV			
**Men B**				Men B	Men B			Men B			
**Men ACWY**						Men ACWY					Men ACWY
**HPV**										HPV	
Vaccinations not actively offered and not free of charge											
**Influenza**				2 doses if not immunized before						1 dose	
**Rotavirus**		RV									
**HAV**						2 doses from 1 year of age					

Influenza, rotavirus and Hepatitis A vaccination (HAV) vaccines were excluded in assessment of vaccination completion as they are not actively offered and are not free of charge.

### Statistical analysis

A descriptive analysis was performed to assess number of vaccines administered in SIS-PED and overall in the Padua region, motivations for SIS-PED admission, vaccine type and any associated acute adverse events. Time trends for the most requested vaccinations and related reasons for referral were also assessed. Results are summarized using frequencies and percentages for qualitative variables and median and standard deviation for continuous variables. Comparisons of categorical variables over time or based on SIS-PED admission was carried out using the chi-square test.

## Results

Three-hundred and fifty-nine children attended the SIS-PED and 560 vaccine doses were administered between 2002 and 2015. Comparing the number of vaccines administered at the SIS-PED with those administered in the Padua Health District, SIS-PED vaccination was only a small percentage of overall vaccination in the region (560/2,127,785 (0.03%)).

### Admission criteria

**Fig 2:**
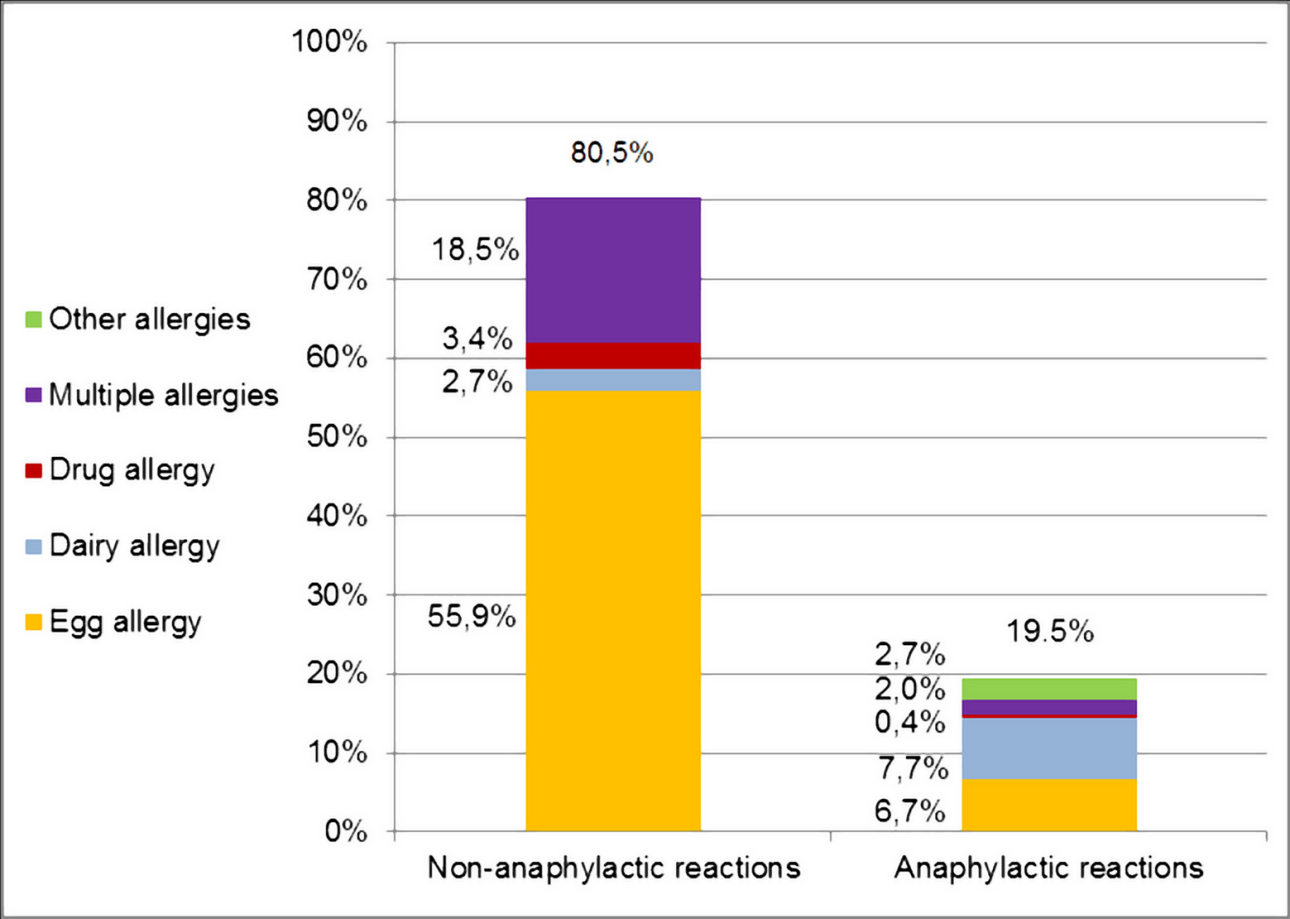
SIS-PED admissions for allergies.

Allergies accounted for 53.0% (297/560) of SIS-PED admissions, 58/297 (19.5%) anaphylactic reaction and 239/297 (80.5%) non-anaphylactic. 186/560 (33.2%) SIS-PED admissions and 166/239 (69.5%) of non-anaphylactic allergy related admissions were for egg allergy, 31/560 (5.5%) were related to dairy allergy, 11/560 (2.0%) to drug allergies, and 59/560 (10.5%) to multiple allergies (Fig 2).

AEs after previous vaccinations accounted for 27.2% (152/560) of SIS-PED admissions. 58/152 (38.2%) of these were immediate IgE mediated reactions, including important local reactions (7/58, 12.1%), urticarial rash or other spread cutaneous reactions (37/58, 63.8%), and bronchospasm (7/58, 12.1%); 16/152 (10.5%) were immediate non-IgE mediated reactions such as vasovagal reactions (3/20, 15%) and hypotonic hypo-responsive episodes (13/20, 65.0%). 78/152 (51.3%) reactions occurred more than two hours after vaccination, most commonly cutaneous reactions (37/74, 50.0%), irritability and/or drowsiness and hypotonia (16/74, 21.6%), and febrile seizures (19/74, 25.6%).

Other reasons for referral accounted for 19.8% (111/560) of SIS-PED admissions. Included in this group were parents who did not wish to vaccinate their children, and children with particular diseases such as neuromuscular disorders, cutaneous mastocytosis, thrombocytopenia, epilepsy, and rare genetic syndromes.

**Fig 3:**
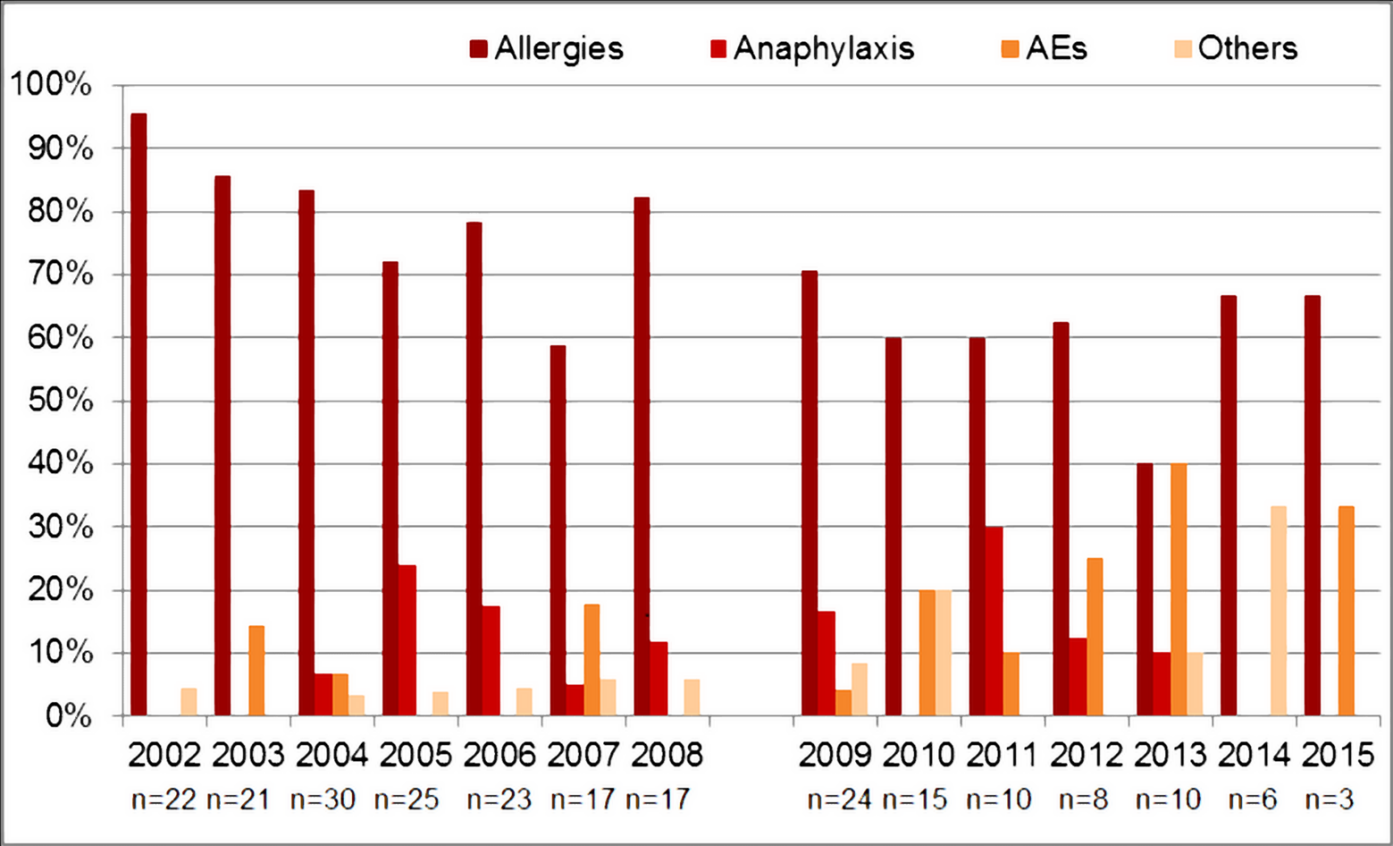
SIS-PED admission criteria for administration of MMR/MMRV vaccines.

From 2009, egg allergy was no longer considered an indication for referral to the SIS for MMR/MMRV vaccination [6,15,16]. Accordingly, 70.0% (30/43) of SIS referrals were for egg allergy in 2004, with only 20.0% (3/16) in 2015 (55.6% for period before 2008 vs 27.0% for period after 2008, p<0.001). Over time the proportion of SIS referrals for anaphylactic allergies remained stable (10.3% before 2008 vs. 11.8% after 2008, p = 0.7) and for AEs increased (Fig 3).

### Administered vaccines

**Fig 4:**
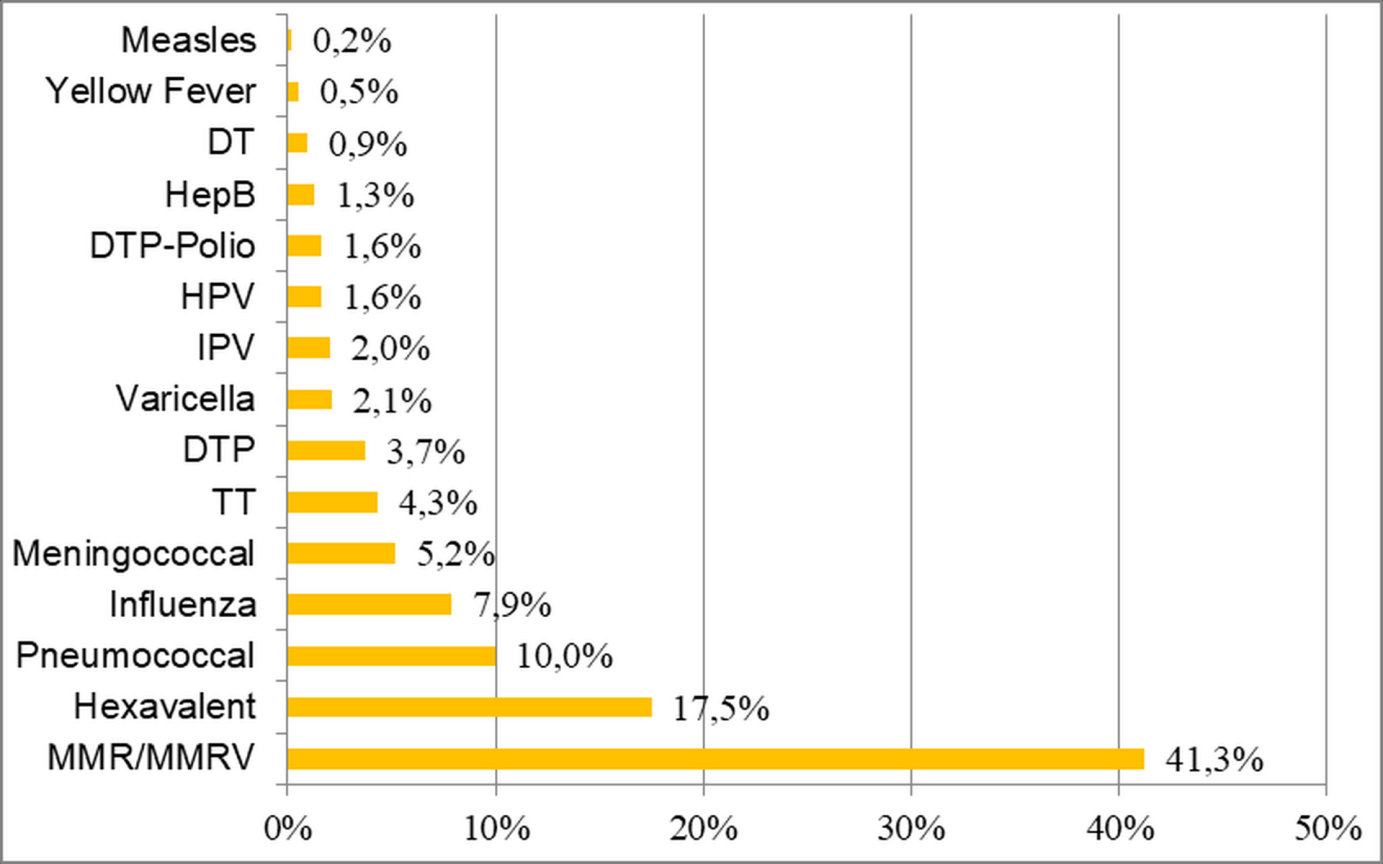
Types of vaccines administered at Padua SIS-PED, 2002–2015.

The most requested vaccination in the SIS-PED was MMR/MMRV (231/560, 41.3%), followed by hexavalent vaccine (98/560, 17.5%). Other vaccinations were requested at a significantly lower rate (Fig 4).

### Adverse events

No serious vaccine-related AEs were observed over 14-years, with 15 mild vaccine-related AEs (15/560, 2.7%).

The vaccinations related to these mild AEs were MMR/MMRV (8/15, 53.3%), hexavalent (3/15, 20.0%), influenza (3/15, 20.0%) and HPV (1/15, 6.7%).

Eight children experiencing mild adverse events after SIS-PED vaccination (8/15, 53.3%) had been referred to the SIS for a previous vaccine-related AE, with six (6/15, 40.0%) referred for allergies. No vaccine-related AEs were recorded among children referred to SIS for parental concerns regarding vaccination.

### Immunization coverage among referrals

All 417 children who attended a pre-vaccination visit at SIS-C were followed up for vaccine schedule adherence, whether they had been admitted for immunization at the SIS-PED or not.

Fifty-eight children (58/417, 13.9%) attending SIS-C were not referred to SIS-PED: 13/417 (3.1%) following recommendation of SIS-C pediatrician to suspend the vaccine plan (11/13 because of a neurologic disease under investigation and 2/13 for previous severe IgE mediated reactions) and 45/417 (10.8%) following recommendation of SIS-C pediatrician to continue vaccination plan with the Health District due to lack of identified risk for vaccine-related AEs. In this case, 25/45 (55.5%) completed their immunization schedules and 20/45 (44.5%) did not.

Among patients vaccinated at SIS-PED, 65/359 (18.1%) continued the vaccination schedule at the Service and 294/359 (81.9%) received only one vaccination at the SIS-PED.

**Fig 5:**
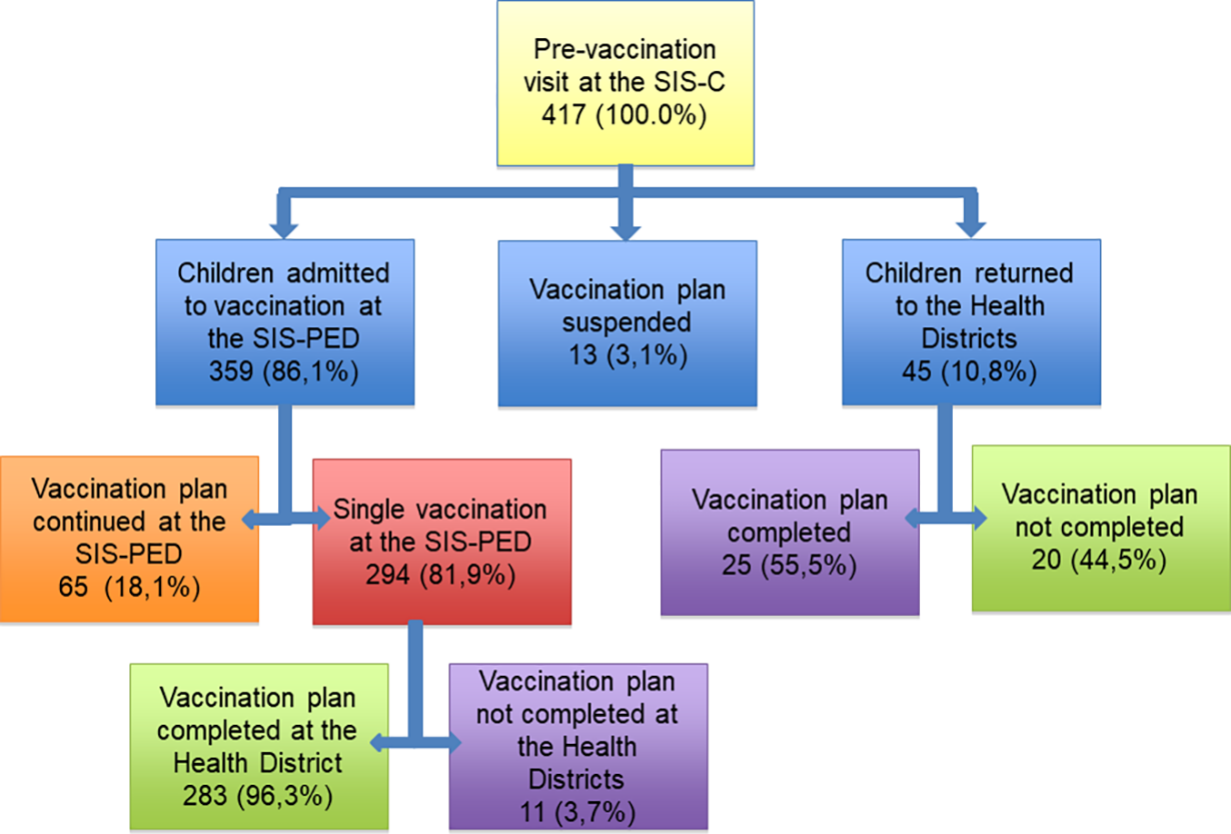
Follow-up of vaccination plan of referrals to Padua SIS-PED, 2002–2015.

Two-hundred eighty-three children (283/294, 96.3%) receiving a single vaccination at SIS-PED completed their vaccination plan at the Local Health District, while 11/294 (3.7%) interrupted the vaccinations for various reasons, including parental refusal. This percentage of completed vaccination plans is significantly higher than the 55.6% (25/45) observed among children receiving a SIS-C recommendation to continue their plan at the Local Health Districts without receiving a single vaccination in SIS-PED (p<0.001) (Fig 5).

## Discussion

Concerns related to the safety of vaccines are increasing, and communication about relative risks and benefits is needed when advising parents on childhood vaccinations. Parents of children who experienced an AE after a previous vaccination, or who have a serious history of anaphylaxis may have particular difficulty in making the decision to complete their child’s vaccination plan. There is a paucity of information about the risk of subsequent vaccine-related AEs, and not all risk factors are completely understood. A SIS may help to address the need for more information, as indicated by increasing participation during the first three years of our service in Padua, Italy.

One important consideration is the feasibility of extending SIS services to a broader segment of the population. From 2002 to 2014, 0.05% to 0.01% of vaccinations in the Padua Health Districts and SIS combined were administered at SIS-PED due to strict admission criteria.

Although utilization of the SIS in Padua remained fairly constant during our study, reasons for referrals to the service varied. Admissions for non-anaphylactic allergies decreased (from 54.5% before 2008 to 31.3% after 2010), likely due to changes in recommendations for children with egg allergies, while admissions for anaphylaxis remained stable around 10%. Referrals for a past history of AE following a previous immunization increased over time, reaching 62.5% in 2015.

We observed very few cases (86/560, 15.4%) of appropriate reason for referral to the SIS, such as an immediate reaction to a previous dose of the same vaccine (presumably IgE-mediated, non-anaphylactic) and a past medical history of anaphylaxis, but not related to any component of the vaccine [6,14].

In our study, 38.8% of admissions with a past history of AE had an immediate IgE mediated reaction, while 10.5% were referred after immediate non-IgE mediated event, such as vasovagal reactions or hypotonic hyporesponsive episodes, and the remaining 51.3% after a delayed reaction, which may be of immunological and non-immunological type [17]. In contrast, only a small number of children were admitted for anaphylaxis. It is a rare event, as reported in a study involving North American children and teenagers where the incidence of anaphylactic reaction was 0.75 cases /1000 person years [18].

Only a few mild AEs after vaccination in SIS-PED were observed in our 14-year experience, confirming the high safety profile of vaccines and the low risk for complications.

We observed a small number of children (2.7%) who experienced minor reactions to the vaccine administration. All were local or systemic reactions and quickly resolved, and all occurred among children referred to SIS for an appropriate reason. These types of vaccine-related AEs are well described in the immunization literature, with a reported incidence around 10% for local reactions and around 5–15% for systemic ones. In addition, the incidence of AEs after revaccination at our SIS-PED was similar to that reported in other studies [11,19]. The Padua SIS-PED may prove useful in assessing and ensuring post-marketing vaccine safety among vaccination subsequent to a vaccine-related AE.

The difference in the proportion of children who completed their vaccination plan at the Health District after just a single vaccination at the SIS-PED (96.3%) and those who completed the plan at the Health District after a SIS-C visit not followed by a SIS-PED admission (55.5%) indicates a potential role of the SIS in promoting the immunization. The SIS addressed understanding the needs of vaccine hesitant parents and improving communication skills and interaction between parents and health care workers. Useful strategies from our SIS included solicitation of questions about vaccines, establishment of a trusting relationship, and provision of appropriate educational materials to parents [20].

The SIS became more important after Regional Decree n.7 of March 23^rd^ 2007 suspended mandatory vaccinations in the Veneto Region from 2008 onwards [10].

More and more frequently parents feel hesitant to vaccinate their children because of conflicting information from media, internet and other sources and were constantly influenced by their social group and anti-vaccination movements [21,22]. Subsequent increases in vaccination refusal may have played a role in a recent measles outbreak in our country [23].

In response Italy's parliament has given final approval to a new program making vaccinations compulsory for school children up to age 16 [24]. For this reason, especially in the immediate future, a continuous updating of the medical staff involved in administering immunizations would be useful to provide appropriate information to families, in order to increase population awareness about this key topic of public health.

## Conclusions

The high percentage of vaccination plans completed among children receiving at least one immunization at the Padua SIS over 14 years suggests a positive influence of the service on intention to vaccinate among concerned parents. A very small number of mild vaccine-related AEs were observed, with no moderate or severe vaccine-related AEs, and all children with an appropriate reason for SIS referral were successfully vaccinated. Specialized immunization services show potential for improving vaccination compliance, increasing immunization coverage and ensuring safe vaccination in high-risk children.

## Supporting information

S1 File Database. (XLSX)
